# 23688 Impact of moving to a virtual format with the Wake Forest School of Medicine (WFSM) Mentor Academy (MA)

**DOI:** 10.1017/cts.2021.560

**Published:** 2021-03-30

**Authors:** Debra I. Diz, Claudia Olivier, Anna Perry, Doug Easterling

**Affiliations:** Wake Forest School of Medicine

## Abstract

ABSTRACT IMPACT: The Wake Forest School of Medicine Mentor Academy has adapted to provide continued effective and relevant formal mentoring training to translational researchers in a virtual format, to improve mentoring and provide effective mentor-mentee communication tools. OBJECTIVES/GOALS: To determine whether the WFSM Mentor Academy (MA), an effective long-standing mentoring program for research faculty, is compromised after moving from an in-person to an online format as a result of COVID-19 restrictions. METHODS/STUDY POPULATION: A vetted National Research Mentoring Network (NMRN) implemented at WFSM addresses 6 major competencies (Effective Communication, Aligning Expectations, Assessing Understanding, Addressing Equity/Inclusion, Fostering Independence, Promoting Professional Development) over 6 months with 10 sessions (20 contact hrs). COVID-19 required that the MA (13 participants) move to an online format after 3 (out of 10) in-person sessions. We survey 26 self-rated mentoring competencies pre- and post MA, based on a numerical 7-point scale (abstract published for ACTS 2020) and, in 2020, included additional assessments of online versus in-person MA satisfaction/effectiveness and perceived impact on abilities of MA participants to mentor in an exclusively virtual format. RESULTS/ANTICIPATED RESULTS: All 13 participants responded to the survey and rated the online format as effective (9) or somewhat effective (4) for learning content. However, for participant interactions, only 4 found it effective and 9 somewhat effective. When assessing ability to mentor in a virtual format, most negatively affected competencies were ‘helping your mentee network effectively’ (7 of 13), ‘motivating your mentee’ (7), and ‘identifying and accommodating different communication styles’ (6). Goal setting (research goals, career goals) was rated easier under COVID-19 restrictions by 3 mentors. Increases in Pre-Post self-expressed mentoring effectiveness (+1 pt quality; +1 pt meeting mentee expectations) are similar to historical values, and 12 of the 13 mentors changed mentoring practices based on MA experiences. DISCUSSION/SIGNIFICANCE OF FINDINGS: While 2020 ratings for increased effectiveness are similar to prior years, since the 2021 MA will remain online, we will adjust content to address challenges identified in training mentors and in mentoring trainees in virtual settings by strategies to keep MA participants engaged online and sharing new resources for virtual/hybrid format mentoring.

